# Molecular Cut-off Values for Aliarcobacter butzleri Susceptibility Testing

**DOI:** 10.1128/spectrum.01003-22

**Published:** 2022-07-07

**Authors:** Quentin Jehanne, Lucie Bénéjat, Astrid Ducournau, Emilie Bessède, Philippe Lehours

**Affiliations:** a French National Reference Center for Campylobacters and Helicobacters, Bordeaux Hospital University Center, Bordeaux, France; b University Bordeaux, INSERM, UMR1312 Bordeaux Institute of Cancer, BRIC, Bordeaux, France; University at Albany, State University of New York

**Keywords:** *Aliarcobacter*, NGS, antimicrobials, susceptibility

## Abstract

Aliarcobacter butzleri is an emerging gastrointestinal pathogen found in many countries worldwide. In France, it has become the third most commonly isolated bacterial species from the stools of patients with intestinal infections. No interpretative criteria for antimicrobial susceptibility testing have been proposed for A. butzleri, and most strains are categorized using the recommendations of the Clinical and Laboratory Standards Institute or the European Committee on Antimicrobial Susceptibility Testing for Campylobacter or *Enterobacterales*. In the present study, the genomes of 30 resistant A. butzleri isolates were analyzed to propose specific epidemiological cut-off values for ampicillin, ciprofloxacin, erythromycin, and tetracycline. The identification of a β-lactamase and the T85I GyrA mutation associated with ampicillin and ciprofloxacin resistance, respectively, allowed us to adjust the disk diffusion (DD) and MIC cut-off values for these molecules. However, epidemiological cut-off values for erythromycin and tetracycline could not be estimated due to the absence of known resistance mechanisms. The present study paves the way for building a consensus for antimicrobial susceptibility testing for this concerning pathogen.

**IMPORTANCE** Aliarcobacter butzleri is an emerging and concerning intestinal pathogen. Very few studies have focused on this particular species, and antimicrobial susceptibility testing (AST) is based on methods that have been mostly developed for Campylobacter spp. In fact, no disk diffusion and E-tests adapted cut-offs for A. butzleri are available which leads to misinterpretations. We have shown here that NGS approach to identify genes and mutations in close relation to phenotypic resistance levels is a robust way to solve that issue and precisely differentiate WT and NWT A. butzleri isolates for frequently used antimicrobials. MIC and DD cut-off values have been significantly adjusted and answer the need for a global consensus regarding AST for A. butzleri.

## INTRODUCTION

Aliarcobacter butzleri (formerly Arcobacter butzleri [1, 2]) is an emerging and concerning intestinal pathogen that was the fourth leading cause of Campylobacter-associated bacterial gastrointestinal infection in 2020 in France after Campylobacter jejuni, C. coli, and C. fetus and is the third most common bacteria isolated from stools (3, 4). A. butzleri has also been associated with bacteremia in immunocompromised patients, including those with cancer or diabetes. Very few studies have focused on this particular species, and antimicrobial susceptibility testing (AST) is based on methods that have been mostly developed for Campylobacter spp. The lack of specific recommendations, such as those proposed by the Clinical and Laboratory Standards Institute (5) (CLSI) or the European Committee on Antimicrobial Susceptibility Testing (6) (EUCAST), leads to the use of different clinical breakpoints and epidemiological cut-off (ECOFF) values recommended for multiple organisms. Therefore, antimicrobial resistance (AMR) studies tend to show inconsistent results.

Many studies, mainly focusing on animal isolates, have reported resistance rates for the most commonly used antimicrobials (7). In particular, bacteria isolated from animal, water, or environmental samples have been shown to be resistant to ciprofloxacin with a rate ranging from 12.5% to 55.8% (8–10). The ampicillin resistance rate is also concerning, representing on average over 50% of the studied isolates. However, while it has been shown that resistance rates can reach extreme values in some countries, such as Ireland (11), erythromycin and gentamicin resistance rates remain largely below 20% globally, and in some cases, no resistance has been reported (12). This lack of resistance among A. butzleri isolates is also observed for tetracycline (13–15). Fewer studies have focused on human samples, and the results have also shown highly variable results regarding erythromycin and tetracycline resistance. In fact, while high rates of resistance have been identified in two studies in Belgium (16, 17), with rates of approximately 80% and 59% to 100%, respectively, recent studies have highlighted high susceptibility rates for these two antimicrobials (18, 19). Ciprofloxacin and gentamicin resistance rates also remain relatively low, at less than 10% and 0%, respectively (16, 18). High ampicillin resistance rates have, however, been observed in the same previous studies, with resistance rates ranging from 79% to 97% (16, 17). Globally, tetracycline, fluoroquinolone, and macrolides are frequently considered appropriate antimicrobials, particularly for intestinal infections (20, 21).

A reliable modern approach to refine susceptibility testing is the use of next-generation sequencing (NGS) and the *in silico* detection of antimicrobial resistance (AMR) mutations and genes. Recent studies have reviewed and analyzed relations between *Aliarcobacter* resistance phenotypes and genotypes (22, 23). Notably, it has been shown that *in vitro* fluoroquinolone resistance (especially ciprofloxacin) is correlated with the presence of specific mutations in the QRDR region of the GyrA protein in positions 85 and 89 (mostly Thr-85-Ile) (24), which are also commonly found among Campylobacter spp., in positions 86 and 90 (25). Similarly, the presence of β-lactamases such as *bla*_OXA-61_ and some versions of the *tet* gene, such as *tet(O)*, *tet(W)*, or *tet(A)*, are responsible for ampicillin and tetracycline resistance, respectively (26, 27). The analysis of a set of efflux pumps (EPs) among a collection of resistant isolates also showed that a particular regulator, TetR, may be involved in erythromycin resistance depending on its protein sequence size (22). The chloramphenicol acetyltransferase (*cat*) gene can also be responsible for chloramphenicol resistance (27).

There is an essential need for accurate interpretative cut-off values specific for A. butzleri susceptibility testing (28). Therefore, in this study, we aimed to (i) estimate resistance profiles using C. jejuni and C. coli EUCAST ECOFF recommended values (6); (ii) identify related genomic resistance mechanisms based on the analysis of A. butzleri clinical isolates from French cases between 2014 and 2016 using NGS for 30 isolates and PCR screening for 71 supplementary isolates; and (iii) estimate specific cut-off values (CO_WT_) for A. butzleri, which could be proposed to national organizations.

## RESULTS

### Antimicrobial susceptibility profiles.

**FIG 1:**
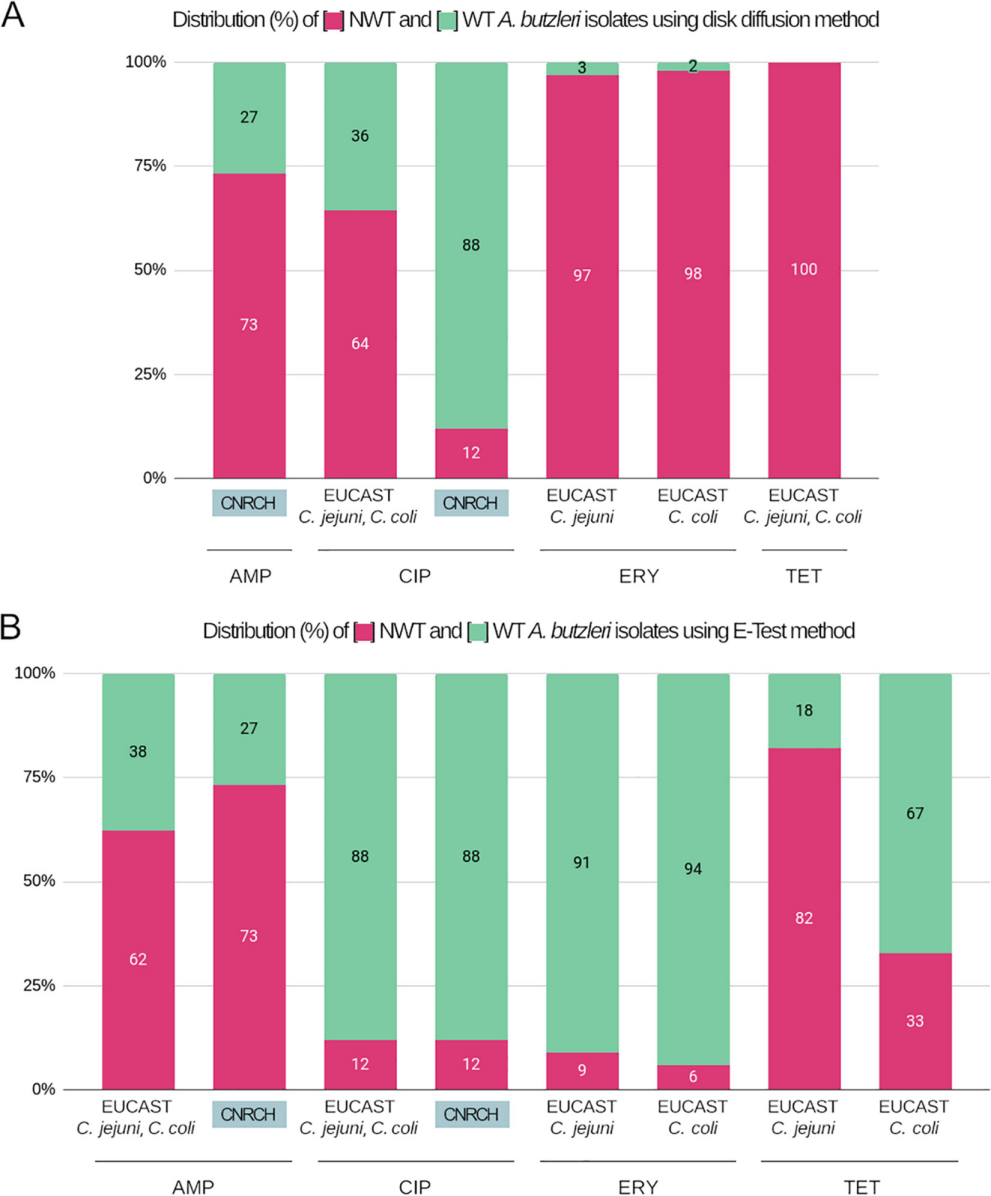
Distributions of percentages of WT and NWT isolates using disk diffusion and Etest methods with recommended EUCAST and adjusted cut-offs. In this study, resistance rates using disk diffusion (A) and Etest (B) were estimated for the following four antimicrobials: ampicillin (AMP), ciprofloxacin (CIP), erythromycin (ERY), and tetracycline (TET). The colors used were as follows: green for WT isolates and red for NWT isolates. EUCAST C. jejuni and C. coli: data interpreted according to the EUCAST ECOFF values proposed for these two species; CNRCH: data interpreted according to the cut-off values (CO_WT_) proposed in the present study.

Considering the C. jejuni and C. coli EUCAST MICs recommendations for ampicillin (Table 1), an overall 62% of A. butzleri isolates were found as non-wild-type (NWT) (*n* = 63 isolates) (Fig. 1) and 38% as wild-type (WT) (*n* = 38). Rates for ciprofloxacin were variable depending on DD or MIC cut-offs. When using DD cut-off values, a total of 64% of isolates were found as NWT (*n* = 65) and 36% WT (*n* = 36). The results obtained when considering ciprofloxacin MIC cut-offs showed completely opposite rates, with 88% WT and 12% NWT. Considering EUCAST DD cut-off values for erythromycin for C. jejuni and C. coli, high rates of NWT isolates were found with, respectively, 97% and 98% of A. butzleri isolates (*n* = 98 and *n* = 99 isolates). In contrary, when using MIC cut-off values for these two Campylobacter species, only 6% to 9% of A. butzleri isolates were found to be NWT, respectively. Finally, various rates were also obtained when AST was performed for tetracycline: all isolates were identified as NWT from DD ECOFF values against 82% and 33% when using MIC ECOFF values defined for C. jejuni and C. coli, respectively.

**TABLE 1:** EUCAST MIC and DD recommended epidemiological cut-off values

		DD (mm)		MIC (mg/L)	
Antimicrobial^*a*^	Species	WT	NWT	WT	NWT
AMP	C. jejuni and C. coli			≤16	>16
CIP	C. jejuni and C. coli	≥26	<26	≤0.5	>0.5
ERY	C. jejuni	≥22	<22	≤4	>4
	C. coli	≥24	<24	≤8	>8
TET	C. jejuni	≥30	<30	≤1	>1
	C. coli	≥30	<30	≤2	>2

a EUCAST recommendations for C. jejuni and C. coli for the following antimicrobials: ampicillin (AMP), ciprofloxacin (CIP), erythromycin (ERY), and tetracycline (TET).

### Identification of resistance mechanisms.

**FIG 2:**
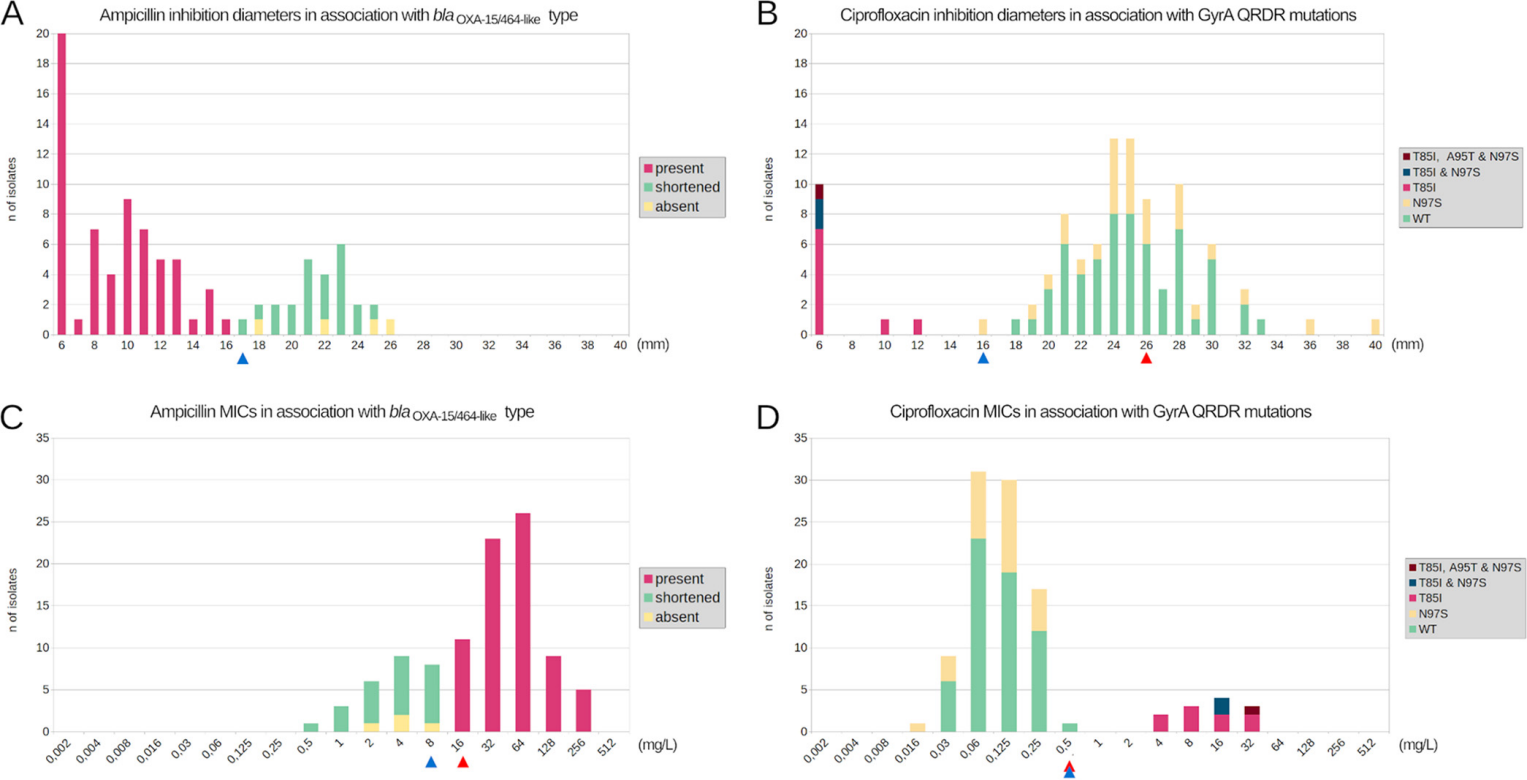
Distributions of ampicillin and ciprofloxacin diameters and MICs according to the resistance mechanism identified for each molecule. Resistance markers are displayed in red or blue, and nonsignificant markers or WT isolates are displayed in yellow or green. Specifically, ampicillin resistance (A and C) is associated with the presence of a *bla*_OXA-15/464-like_ gene (OM617734), and ciprofloxacin resistance (B and D) is associated with the presence of a single mutation (T85I) in the GyrA protein sequence (OM617736). Moreover, red triangles represent EUCAST epidemiological cut-off values for C. jejuni and C. coli and blue triangles represent CO_wt_ values proposed in the present study.

The genomes of 30 A. butzleri isolates were analyzed using NGS. As displayed in Fig. S1, genome sizes were on average 2.29 Mbp in length (s.d. ± 109 Kbp) (GC% of approximately 28), consistent with previously published A. butzleri genome lengths (29) estimated to be ≃ 2.3 Mbp, and the average numbers of contigs and coding DNA sequences (CDS) were 40 (of ≃ 59 Kbp average size) and 2,268, respectively. Species identification using ANI revealed that all isolates were significantly positive (⩾95%) to A. butzleri species, with an average score of 97.4% (s.d. ± 0.37%). The determination of antimicrobial resistance markers showed the presence of a *bla*_OXA-15/464-like_ gene (22) in two different versions, full gene (OM617734) or shortened sequence (OM617735), detected within the genome of all 30 sequenced isolates (Table 2): a total of 20 isolates (67%) with high AMP MIC and low DD values displayed the full gene version and; on the contrary, 10 isolates (33%) with low MIC and high DD values possessed a half-size shortened sequence of *bla*_OXA-15/464-like_ due to large deletion of the first 396 nucleotides. Using PCR screening, 54 supplementary isolates (76%) displayed the *bla*_OXA-15/464-like_ gene, and 17 isolates (24%) displayed the shortened sequence or none. Moreover, the presence of the β-lactamase in its full version induced a 15-fold increase in AMP MIC, from 4.3 (±2.7) to 67.7 (±60.7) mg/L on average, and a 2.5-fold decrease in inhibition diameters, from 21.7 (±2.3) to 8.8 (±2.9) mm on average (Fig. 2A, C), clearly indicating that the deletion may lead to the protein inactivation.

**TABLE 2:** List of all 101 A. butzleri clinical isolates used in this study^*a*^

Isolate^*b*^	WGS^*b*^	Accession	Sex	Age	Inhibition zone diameters (mm)^*c*^				MICs (mg/L)^*c*^					Corresponding resistance markers		
					AMP	CIP	ERY	TET	AMP	CIP	ERY	TET	AUG	*bla*_OXA-15/464_ ^*d*^	GyrA QRDR mutation^*d*^	TetR protein^*d*^
2014-3116	Yes	JAKKPY000000000	F	71	6	6	15	22	64	8	4	2	12	Present	T85I	Conserved
2014-3403	Yes	JAKKPX000000000	F	82	10	24	13	18	64	0.125	4	2	4	Present	N97S	Conserved
2015-0036	Yes	JAKKPW000000000	M	65	13	25	15	21	32	0.125	2	2	4	Present	N97S	Conserved
2015-0045	Yes	JAKKPV000000000	M	69	21	32	19	28	4	0.06	2	1	0.38	Shortened	WT	Conserved
2015-0097	Yes	JAKKPU000000000	M	72	22	29	19	18	8	0.125	1	2	0.75	Shortened	WT	Conserved
2015-0120	Yes	JAKKPT000000000	M	69	21	28	18	22	4	0.06	2	2	1.5	Shortened	WT	Conserved
2015-0463	Yes	JAKKPS000000000	M	44	23	29	16	22	1	0.06	2	1	0.19	Shortened	N97S	Conserved
2015-0489	Yes	JAKKPR000000000	M	60	6	21	16	19	256	0.125	4	4	16	Present	WT	Conserved
2015-0654	Yes	JAKKPQ000000000	M	98	10	28	24	24	32	0.125	1	1	3	Present	WT	Conserved
2015-1201	Yes	JAKKPP000000000	M	7	21	25	17	22	1	0.06	2	2	1	Shortened	WT	Conserved
2015-1220	Yes	JAKKPO000000000	F	50	6	33	15	20	256	0.25	16	4	16	Present	WT	Conserved
2015-1650	Yes	JAKKPN000000000	M	81	17	12	18	24	4	4	4	2	0.75	Shortened	T85I	Conserved
2015-185H	Yes	JAKKPM000000000	F	94	6	6	14	19	64	8	4	1	12	Present	T85I	Conserved
2015-2363	Yes	JAKKPL000000000	F	53	6	6	15	20	64	16	2	4	6	Present	T85I & N97S	Conserved
2015-2485	Yes	JAKKPK000000000	M	34	6	24	17	18	32	0.125	4	2	8	Present	N97S	Conserved
2015-2901	Yes	JAKKPJ000000000	F	2	19	28	18	19	2	0.03	1	1	0.75	Shortened	WT	Conserved
2016-0341	Yes	JAKKPI000000000	F	28	6	20	15	18	128	0.125	4	4	16	Present	WT	Conserved
2016-0474	Yes	JAKKPH000000000	M	58	24	23	13	22	1	0.125	16	4	0.25	Shortened	WT	New
2016-0547	Yes	JAKKPG000000000	F	88	23	22	13	20	8	0.25	16	1	1	Shortened	WT	Truncated
2016-1192	Yes	JAKKPF000000000	M	72	6	28	12	18	64	0.06	4	4	8	Present	N97S	Conserved
2016-2353	Yes	JAKKPE000000000	M	80	23	6	18	19	4	32	1	4	1	Shortened	T85I, A95T & N97S	Conserved
2016-23H	Yes	JAKKPD000000000	NA	NA	6	28	17	17	128	0.06	2	4	8	Present	WT	Conserved
2016-2642	Yes	JAKKPC000000000	M	65	6	25	15	20	128	0.125	2	2	12	Present	WT	Conserved
2016-2978	Yes	JAKKPB000000000	M	73	10	32	20	24	32	0.03	2	1	6	Present	N97S	Conserved
2016-3169	Yes	JAKKPA000000000	M	64	6	6	15	20	256	32	2	2	16	Present	T85I	Conserved
2016-3175	Yes	JAKKOZ000000000	M	80	6	6	18	21	64	16	4	4	12	Present	T85I & N97S	Conserved
2016-3218	Yes	JAKKOY000000000	F	50	6	24	15	20	256	0.125	2	2	12	Present	N97S	Conserved
2016-3224	Yes	JAKKOX000000000	F	82	16	30	21	23	16	0.03	4	2	3	Present	WT	Conserved
2016-3393	Yes	JAKKOW000000000	F	67	6	28	17	19	128	0.06	2	2	12	Present	N97S	Conserved
2016-3396	Yes	JAKKOV000000000	F	3	6	25	15	20	128	0.06	0.125	2	12	Present	WT	Conserved
2014-2744	No		M	69	6	24	14	18	64	0.25	4	2	4	Present	WT	No WGS & PCR
2014-2752	No		M	19	23	25	14	18	8	0.06	2	4	1	Shortened	WT	No WGS & PCR
2014-2753	No		M	61	10	23	14	21	32	0.125	1	2	1.5	Present	WT	No WGS & PCR
2014-2827	No		M	76	13	6	13	18	16	32	1	2	3	Present	T85I	No WGS & PCR
2014-2856	No		M	81	10	40	15	24	32	0.03	2	1	6	Present	N97S	No WGS & PCR
2014-2861	No		M	81	6	18	10	19	128	0.5	16	4	8	Present	WT	No WGS & PCR
2014-2964	No		F	48	21	22	10	19	4	0.25	8	2	2	Shortened	WT	No WGS & PCR
2014-2991	No		M	2	23	20	13	21	4	0.25	8	2	1.5	Shortened	WT	No WGS & PCR
2014-3221	No		M	64	13	36	18	26	16	0.016	1	0.5	2	Present	N97S	No WGS & PCR
2014-3250	No		F	83	18	21	13	20	8	0.125	2	2	1.5	Absent	WT	No WGS & PCR
2014-3252	No		F	71	21	26	13	21	2	0.03	2	2	1	Shortened	WT	No WGS & PCR
2014-3700	No		M	1	13	21	14	21	16	0.06	4	2	6	Present	WT	No WGS & PCR
2014-3763	No		M	1	8	20	14	20	64	0.25	4	4	8	Present	WT	No WGS & PCR
2015-0185	No		M	80	11	32	17	22	64	0.06	2	2	6	Present	WT	No WGS & PCR
2015-0245	No		M	65	15	30	24	27	32	0.06	1	1	6	Present	WT	No WGS & PCR
2015-0265	No		M	10	26	25	20	19	4	0.06	2	2	1.5	absent	WT	No WGS & PCR
2015-0919	No		F	2	6	16	15	18	64	0.25	4	4	8	Present	N97S	No WGS & PCR
2015-0922H	No		F	23	6	25	19	22	64	0.06	4	2	16	Present	WT	No WGS & PCR
2015-0963H	No		M	4	6	30	17	21	32	0.125	4	2	6	Present	WT	No WGS & PCR
2015-1073	No		F	77	7	24	10	22	64	0.25	16	4	8	Present	N97S	No WGS & PCR
2015-1078	No		F	84	11	10	19	18	32	4	1	2	8	Present	T85I	No WGS & PCR
2015-1212	No		M	64	9	21	14	19	64	0.125	4	4	12	Present	N97S	No WGS & PCR
2015-1462	No		M	84	19	22	13	19	4	0.125	4	4	2	Shortened	WT	No WGS & PCR
2015-1534	No		M	73	11	26	17	21	32	0.06	2	1	12	Present	N97S	No WGS & PCR
2015-1631	No		F	91	8	24	16	19	64	0.125	4	4	16	Present	WT	No WGS & PCR
2015-2133	No		F	91	6	21	13	17	128	0.25	2	4	6	Present	WT	No WGS & PCR
2015-2224	No		M	78	15	26	19	21	16	0.06	2	2	3	Present	WT	No WGS & PCR
2015-2279	No		F	83	18	24	16	22	8	0.125	1	2	1	Shortened	WT	No WGS & PCR
2015-2573	No		M	36	8	6	16	19	32	16	2	2	4	Present	T85I	No WGS & PCR
2015-2637	No		M	72	22	21	13	18	8	0.125	4	2	1	Shortened	WT	No WGS & PCR
2015-2642	No		F	88	6	24	15	18	128	0.125	2	4	8	Present	WT	No WGS & PCR
2015-2679	No		M	53	8	30	23	23	256	0.03	0.5	1	4	Present	N97S	No WGS & PCR
2015-2704	No		M	87	11	21	16	18	32	0.125	4	2	6	Present	WT	No WGS & PCR
2015-2713	No		M	26	11	27	18	21	32	0.06	2	2	8	Present	WT	No WGS & PCR
2015-2723	No		F	64	20	23	14	21	8	0.125	4	2	2	Shortened	WT	No WGS & PCR
2015-2923	No		M	85	11	19	19	18	16	0.25	4	2	4	Present	N97S	No WGS & PCR
2015-2993	No		F	90	15	23	17	21	32	0.06	2	1	2	Present	WT	No WGS & PCR
2016-0027	No		F	65	9	25	15	22	64	0.06	4	2	8	Present	N97S	No WGS & PCR
2016-0107H	No		F	43	6	28	17	20	64	0.06	4	2	4	Present	N97S	No WGS & PCR
2016-0157	No		F	NA	14	6	16	19	16	8	2	2	2	Present	T85I	No WGS & PCR
2016-0182	No		F	62	23	26	20	21	2	0.06	4	4	0.19	Shortened	WT	No WGS & PCR
2016-0199H	No		F	71	25	25	18	18	0.5	0.03	1	2	0.38	Shortened	WT	No WGS & PCR
2016-0375	No		M	67	8	22	16	17	32	0.25	4	4	4	Present	WT	No WGS & PCR
2016-0404	No		M	25	6	24	16	19	64	0.25	4	4	4	Present	WT	No WGS & PCR
2016-0458H	No		NA	N/A	22	22	11	21	2	0.25	16	2	0.75	Shortened	N97S	No WGS & PCR
2016-0475	No		F	79	6	25	17	19	64	0.125	4	4	12	Present	N97S	No WGS & PCR
2016-0483	No		F	88	9	26	17	18	32	0.125	4	1	6	Present	N97S	No WGS & PCR
2016-0539	No		F	2	22	26	18	20	4	0.06	2	2	1	absent	WT	No WGS & PCR
2016-0549	No		F	65	12	28	21	25	32	0.03	1	1	4	Present	WT	No WGS & PCR
2016-0635	No		F	1	11	28	18	25	16	0.06	4	1	3	Present	WT	No WGS & PCR
2016-0636H	No		F	84	24	23	13	20	2	0.125	2	2	2	Shortened	WT	No WGS & PCR
2016-0898H	No		M	83	13	24	16	22	16	0.06	4	2	2	Present	WT	No WGS & PCR
2016-0924	No		M	66	6	25	18	19	64	0.125	2	0.5	8	Present	N97S	No WGS & PCR
2016-0987H	No		M	67	12	24	16	28	16	0.25	4	4	2	Present	WT	No WGS & PCR
2016-1184	No		M	2	25	26	21	25	2	0.03	2	1	1	absent	WT	No WGS & PCR
2016-1246	No		F	81	9	23	17	19	32	0.06	4	2	6	Present	N97S	No WGS & PCR
2016-1943	No		F	95	8	30	15	22	64	0.06	2	2	6	Present	WT	No WGS & PCR
2016-2313	No		F	69	6	24	13	20	64	0.25	1	4	6	Present	N97S	No WGS & PCR
2016-2366	No		F	77	6	25	13	19	64	0.125	2	4	8	Present	N97S	No WGS & PCR
2016-2421	No		F	56	6	21	16	17	64	0.125	4	4	6	Present	N97S	No WGS & PCR
2016-2439	No		F	66	10	20	13	22	64	0.125	2	2	8	Present	N97S	No WGS & PCR
2016-2571	No		M	62	6	26	21	21	64	0.125	2	4	16	Present	WT	No WGS & PCR
2016-2643	No		M	28	12	27	15	21	16	0.06	1	2	2	Present	WT	No WGS & PCR
2016-2785	No		M	82	10	24	18	19	32	0.25	2	4	4	Present	WT	No WGS & PCR
2016-2786	No		F	71	10	25	12	20	32	0.125	4	4	4	Present	WT	No WGS & PCR
2016-2832	No		F	70	6	19	13	18	128	0.25	2	8	8	Present	WT	No WGS & PCR
2016-2841	No		F	69	12	27	20	20	32	0.06	2	4	4	Present	WT	No WGS & PCR
2016-3051	No		F	79	12	30	17	20	32	0.06	1	2	3	Present	WT	No WGS & PCR
2016-3249	No		M	4	20	28	16	22	8	0.06	1	2	0.75	Shortened	WT	No WGS & PCR
2016-3287	No		F	57	10	26	15	21	64	0.06	8	2	12	Present	N97S	No WGS & PCR
2016-3346	No		M	6	8	6	17	18	32	16	2	4	6	Present	T85I	No WGS & PCR

a Metadata for all clinical isolates analyzed in the present study are shown here.

b Isolates were sorted based on their id and NGS status: 30 isolates were sequenced and used for antimicrobial resistance marker identification (“yes” value, BioSample ids are available in this table), and 71 were used for validation (“no”).

c AST using DD and MIC were performed for all 101 isolates using ampicillin (AMP), ciprofloxacin (CIP), erythromycin (ERY) and tetracycline (TET) (and MIC for amoxicillin+clavulanic acid - AUG), and the corresponding results are shown in mm and mg/L, respectively.

d Finally, associated resistance markers for ampicillin, ciprofloxacin and erythromycin are displayed at the end of the table.

**FIG 3:**
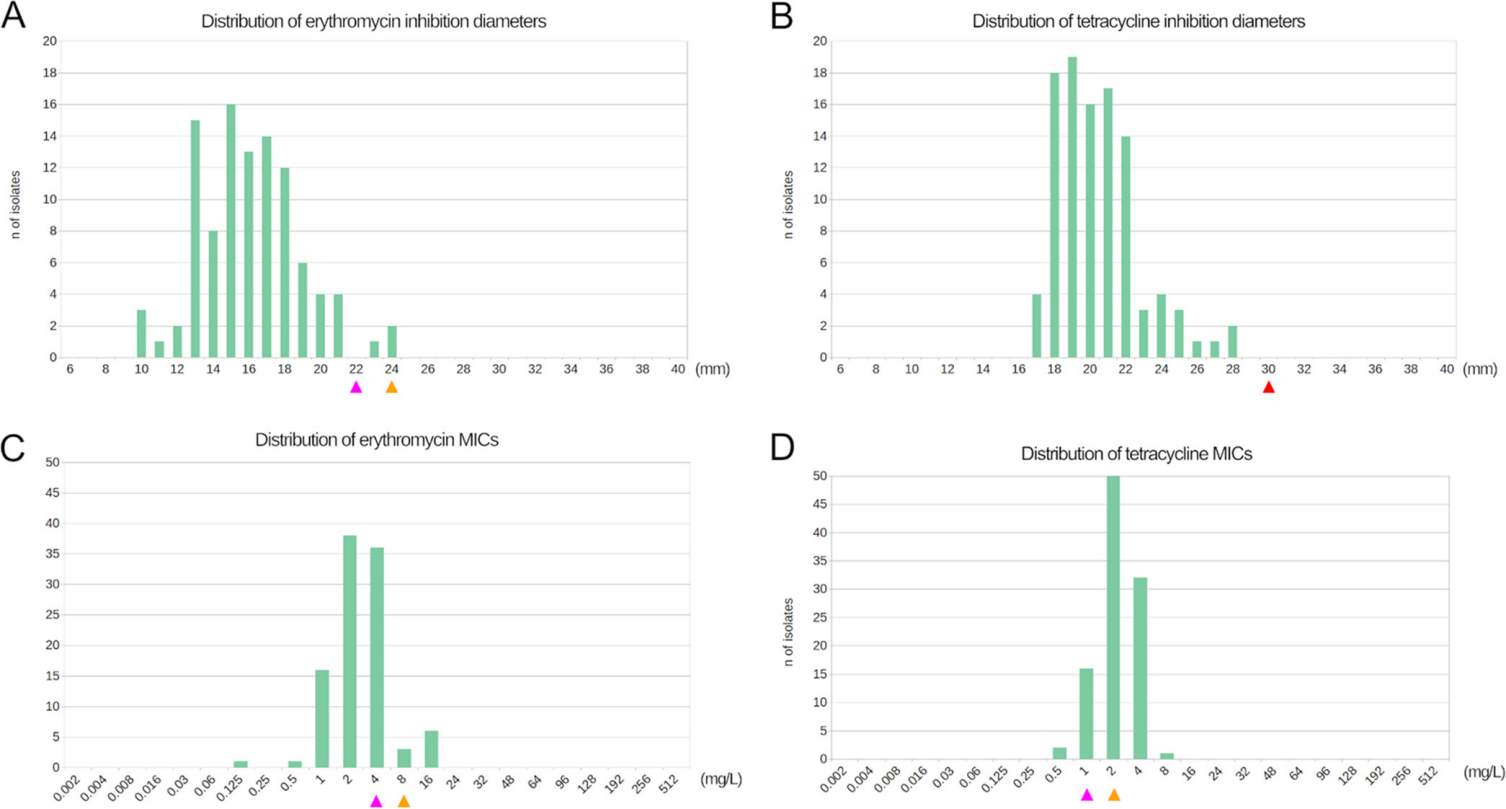
Distributions of erythromycin and tetracycline diameters and MICs. MICs in mg/L (A and C) or DD in mm (B and D) values are displayed here. Pink triangles represent EUCAST epidemiological cut-off values for C. jejuni, orange triangles for C. coli and red triangles for both.

Using WGS, mutation T85I in the QRDR region of GyrA responsible for ciprofloxacin resistance in A. butzleri and various other bacteria, such as Campylobacter and *Helicobacter* (24, 25), was found in 7 NWT isolates (20%) (Table 2) (OM617736). Mutation N97S was also found in 11 NWT isolates (37%), and A95T was found in a unique isolate (2016-2353), but no significant increase in ciprofloxacin MIC was observed. Additionally, a total of three potentially ciprofloxacin resistant isolates (2015-2363, 2016-2353, and 2016-3175) (10%) displayed more than one mutation among T85I, N97S and A95T. Using PCR and sequencing of the *gyrA* sequence from 71 supplementary isolates, the mutation T85I was found in five of them (7%), and the mutation N97S was found in 20 (28%). Overall, the presence of T85I, in contrast to the N97S or WT isolate, has a significant impact on CIP MIC and inhibition diameters. In fact, a 134-fold increase in MIC from 0.1 (±0.08) to 16 (±10.7) mg/L on average, and a 4-fold decrease in inhibition diameters from 25.2 (± 3.9) to 6.8 (± 2.0) mm on average, were observed in NWT isolates (Fig. 2B, D). Finally, no significant resistance mechanism for erythromycin and tetracycline was identified among our collection of A. butzleri isolates. Moreover, distributions of inhibition diameters and MICs confirmed the presence of a single population among our collection of 101 strains (Fig. 3). Therefore, it is unclear that EUCAST cut-offs may or may not be applicable and their use could lead to incorrect resistance rate estimations.

### Adjustment in MICs and DD cut-off values for ampicillin and ciprofloxacin.

Here, we showed that AST using both DD and MIC can lead to strong variability in population proportions (Fig. 1). Combining *in vitro* AST with the *in silico* NGS method, we have shown the presence of a *bla*_OXA-15/464-like_ gene and a mutation in GyrA at position 85 are associated with ampicillin and ciprofloxacin resistance, respectively. Consequently, inhibition diameters and MIC distributions showed two distinct populations of A. butzleri isolates: WT and NWT isolates (Fig. 2A to D). These data result in CO_WT_ values for ampicillin and ciprofloxacin for disk diffusion defined to NWT < 17 mm and NWT < 16 mm, respectively. MIC CO_WT_ value should also be slightly adjusted to NWT > 8 mg/L for ampicillin and stay unchanged for ciprofloxacin, which has also been shown in a previous A. butzleri AST study (28). Considering these A. butzleri specific DD and MIC CO_WT_ values, 73% (*n* = 74) and 27% (*n* = 27) were considered NWT and WT to AMP, respectively, and 12% (*n* = 12) and 88% (*n* = 89) of isolates were considered NWT and WT to CIP, respectively (Fig. 1).

## DISCUSSION

Emerging resistance to antimicrobials is concerning, especially in regard to species that have been rarely studied to date. In this study, we focused our attention on a pathogen closely related to Campylobacter, A. butzleri (30). AST using disk diffusion or E-tests is seriously lacking in sensitivity because of the absence of epidemiological cut-offs adapted for this species, leading to AST misinterpretation. To better differentiate WT and NWT A. butzleri isolates, we here favored the use of NGS to determine genomic resistance markers against four frequently used antimicrobials: ampicillin (AMP), ciprofloxacin (CIP), tetracycline (TET), and erythromycin (ERY). This DNA-based approach allowed us to identify genes and mutations in close relation to phenotypic resistance levels: the T85I mutation in the QRDR region of the gyrase subunit A (OM617736)—already largely described worldwide (24, 25) and the presence of a *bla*_OXA-15/464-like_ gene (OM617734), associated with resistance to AMP and CIP. Moreover, we have shown that the EUCAST MIC and DD cut-off values for ampicillin and ciprofloxacin could be significantly adjusted to A. butzleri species. In fact, CO_WT_ values have been estimated as follows: AMP (NWT) < 17 mm | AMP (NWT) > 8 mg/L and CIP (NWT) < 16 mm | CIP (NWT) > 0.5 mg/L (Fig. 2, blue arrows).

*bla*_OXA-15/464-like_ was first identified as a putative β-lactamase within A. butzleri strain RM4018 in 2007 in the United States (29) and was present in unpublished strains C0903 (KU721147) and B0367 (KU721148) from Scotland in 2016. Recently, its expression was related to ampicillin resistance in Portuguese isolates in 2020 (22). However, the presence of a shortened sequence of this β-lactamase among potentially susceptible isolates has not been mentioned yet in any publication. Moreover, no *bla*_OXA-61_ or any mutation in its promoting region, as commonly found in ampicillin-resistant Campylobacter spp. (31, 32)., was identified in our A. butzleri isolates. This suggests that the mere presence or absence (or shortened sequence) of *bla*_OXA-15/464-like_ modulates ampicillin resistance. Additionally, *bla*_OXA-15/464-like_ positive bacteria tend to maintain high MIC levels for amoxicillin in the presence of clavulanic acid (Fig. S2), suggesting that this *bla*_OXA-15/464-like_ gene is not sensitive to the inhibitory effect of clavulanic acid, as shown in various bacterial species in previous studies, including Campylobacter spp. (33, 34).

Regarding erythromycin resistance, neither a mutation in the 23S rRNA genes sequence (35) nor the presence of methyltransferases such as *erm*(B) and *erm*(N), as described in Campylobacter spp. isolates (36), have been identified. In 2020, Isidro et al. (22) showed that the protein size of the TetR regulator (ABU_RS11100) could be associated with erythromycin resistance. In fact, the alignment of 20 TetR sequences from the present study combined with 17 supplementary sequences from Portugal (22) revealed high MIC values (ERY MIC > 8 mg/L) among isolates with a truncated (OM617733) or new TetR protein sequence (counting two French clinical isolates, JAKKPH000000000 and JAKKPG000000000; Fig. S3). However, due to the low number of isolates with high level of MIC that were analyzed in this study, no clear association could be drawn between TetR protein and erythromycin DD or MIC values. We strongly recommended not to use C. jejuni and C. coli DD or MIC cut-offs for erythromycin AST (NWT < 22 mm | NWT > 4 mg/L or NWT < 24 mm | NWT > 8 mg/L for C. jejuni and C. coli, respectively). In fact, this strategy may lead to incorrect antimicrobial categorization because no distinct WT and NWT populations could be observed (Fig. 3A, C), which is in line with a previous A. butzleri AST study (28). The same results were finally obtained for the identification of tetracycline-resistant profiles using NGS among our set of isolates. Specifically, *tet(O)*, which has been shown to be related to high levels of MIC in A. butzleri (37) and various other species (26, 38), and *adeF* (37) were undetected in our collection. We recommend that EUCAST tetracycline epidemiological cut-off values for C. jejuni and C. coli should not be considered for A. butzleri because no clear WT and NWT population can be distinguished (Fig. 3B, D). Isolates exhibiting high MIC and small DD values will need to be systematically sequenced and analyzed using resistance marker databases. Data from various ecological niches are available (22, 23, 27, 39) and are crucial resources to monitor and compare antimicrobial resistance distributions between most sources of infection.

Finally, the description of A. butzleri as a multiresistant species may likely be overstated (40–42). Clinical breakpoints based on pharmacologic and epidemiological cut-off values can in fact lead to significant mismatches between genomic and phenotype. It is especially true when AST is performed both from DD and MIC, where considerable discrepancies can be observed. In fact, we have shown that AST for ciprofloxacin, erythromycin, and tetracycline can either define A. butzleri isolates as mostly NWT using DD, or mostly WT using MIC (Fig. 1). Globally, AST using Etest showed more accurate associations between genomic resistance markers determination and EUCAST ECOFF values than the disk diffusion method (Fig. 2) and should be considered first while dealing with A. butzleri isolates. Additionally, national guidelines suggest different epidemiological cut-off values for identical antimicrobials, which does not benefit accurate specificity. Here, NGS has revealed gaps between *in vitro* resistant isolates based on standard recommendations and *in silico* identification of antimicrobial resistance markers. This is particularly true when AST is performed using the DD method for erythromycin and tetracycline, where epidemiological cut-off values tend to misinterpret a given isolate as NWT. Based on the fact that most isolates did not display specific resistance markers for erythromycin and tetracycline, the A. butzleri resistance rate for these two antimicrobials may be considered low, similar to previous works (13–15), but in contradiction with others (37, 41, 43). Moreover, the identification of genomic resistance markers for ampicillin and ciprofloxacin has allowed us to obtain more accurate results for these two antimicrobials. Therefore, these A. butzleri-specific CO_WT_ values need to be considered by the EUCAST or CLSI organizations. MIC and DD distributions analyses must still be performed from separate laboratories in order to define these results as valid epidemiological cut-off values (44). In addition, the aggregated distributions will need to contain at least 100 MIC values in the putative WT distribution. The need for a global consensus regarding AST for A. butzleri is high, and the expansion of NGS provides robust ways to solve that issue, especially for less studied species.

## MATERIALS AND METHODS

### A. butzleri isolate selection, culture conditions, and antimicrobial susceptibility testing.

A total of 101 A. butzleri clinical isolates isolated from human stools from French patients between 2014 and 2016 were analyzed in this study (Table 2). This data set consists of most antimicrobial resistant A. butzleri isolates of the French National Reference Center for Campylobacters & Helicobacters (3) from that period of time. The mean age and female percentage of this data set were 59 years old and 47.47%, respectively. Each isolate was recovered from frozen stocks (−80°C in in-house peptone +20% glycerol broth) on Columbia blood agar plates with 5% sheep blood (Thermo Fisher Scientific, MA). Plate incubations were performed at 37°C in jars using an Anoxomat microprocessor (Mart Microbiology, B.V. Lichtenvoorde, the Netherlands), which creates an atmosphere of 80% to 90% N2, 5% to 10% CO2, and 5% to 10% H2, and species were identified using MALDI-TOF mass spectrometry (MS) as previously described (45). Antimicrobial susceptibility testing (AST) of ampicillin (AMP), ciprofloxacin (CIP), tetracycline (TET), and erythromycin (ERY) was performed for 24 h using Mueller–Hinton (MH) agar plates supplemented with 5% defibrinated horse blood (MH-F) and 20 mg/L β-NAD (bioMérieux, Marcy l’Etoile, France) + 0.5 McFarland inoculum, for both disk diffusion (DD) (Bio-Rad, Marnes-La-Coquette, France) and MIC estimations (Etest, bioMérieux). Isolates were classified as WT or NWT based on the EUCAST ECOFF values for C. jejuni and C. coli (6), which are listed in Table 1. Inhibition zone diameters were measured using the SIRscan Auto (i2A, Montpellier, France) automatic system (46), and MICs were read by two independent readers at the position where the zone of growth inhibition intersected the Etest strip. The C. jejuni ATCC 33560 reference strain was used as a quality control strain, according to the EUCAST recommendations.

### Next-generation sequencing and genomic antimicrobial resistance identification.

Initially, 30 multiresistant A. butzleri isolates were selected to perform NGS and genomic resistance marker identification. Bacterial DNA was extracted using the MagNA Pure 6 DNA and Viral NA SV Kit, and purification was performed from bacterial lysis on a MagNA Pure 96 System (Roche Applied Science, Manheim, Germany). Spectrophotometry using NanoDrop Technologies (Wilmington, DE, USA) was performed on all DNA samples for quantification and purity checks (260/280 and 260/230 ratios). Following DNA extraction, NGS was performed using an Illumina HiSeq 4000 machine (Integragen, Evry, France), quality tests were run using FastQC v0.11.9 (47), and raw (.fastq) data were cleaned using Sickle v1.33 (48) and assembled using SPAdes v3.10.1 (49). Species identification of all isolates was also performed using FastANI v1.1 (50) against A. butzleri reference genomes NCTC 12481 (51) and RM4018 (29). The studied genomes are available in the NCBI database under BioProject PRJNA798874, and the corresponding identifiers are presented in Table 2. Finally, the determination of associated antimicrobial genomic resistance markers was performed using Prokka v1.14.6 (52) annotation software and the Comprehensive Antibiotic Resistance Database (CARD) Resistance Gene Identifier webtool (card.mcmaster.ca/analyze/rgi) (53).

### PCR screening and sequencing of antimicrobial resistance markers.

In order to validate computational antimicrobial resistance identifications, primers for the detection of ampicillin- and ciprofloxacin-resistance genomic markers were designed using Primer3 v2.5.0 (54) and tested on a subset of 71 A. butzleri resistant clinical isolates (Table 2, NGS column = “no”). Ampicillin resistance was detected from PCR screening of the *bla_OXA-15/464-like_* conserved (resistant isolate) gene using F1/R1 primers pairs or shortened sequence (susceptible isolate) using the F2/R1 primers pairs. Primers were designed in conserved regions within the gene sequence, as follows: (F1) 5′-ATACCAAGTTGAAGGAAC-3′, (R1) 5′-GTTGGGAAGGAAAATATGG-3′, (F2) 5′-TAGGCAAAGATGTAACTG-3′. Amplifications were performed using PCR program (1) in Table S1 and displayed on 2% agarose gels containing Midori Green Advance coloring (Nippon Genetics Europe, Düren, Germany) with expected product sizes for conserved and shortened *bla_OXA-15/464-like_* of 501 bp and 165 bp, respectively. GyrA QRDR amplification to detect mutations responsible for ciprofloxacin resistance was performed using the following primers: (F1) 5′-TGGATTAAAACCAGTTCATAGAAG-3′, (R1) 5′-GTTCCAAATTATGATGATACGATGA-3′ and PCR program (1), as described by Abdelbaqi et al. (55). Amplified products with an expected size of 344 bp were dyed using a BigDye Terminator v3.1 Cycle Sequencing Kit (Thermo Fisher Scientific, MA) and PCR program (2) in Table S1 prior to sequencing using Applied Biosystems Sanger Sequencing 3500 Series device. Finally, DNA sequences were aligned using MEGAX v10.1.7 software (56).

### Data availability.

The assembled genomes are available under BioProject PRJNA798874 and BioSamples SAMN25131732 to SAMN25131761. The full accession list is provided in Table 2.
