# Supplementary material for: Molecular Cut-off Values for Aliarcobacter butzleri Susceptibility Testing
Source: Microbiol Spectr. 2022 Jul 7;10(4):e01003-22. doi: 10.1128/spectrum.01003-22 (PMC9430808; doi:10.1128/spectrum.01003-22)
Supplement: Supplemental file 1 — Table S1 and Fig. S1 to S3. Download spectrum.01003-22-s0001.pdf, PDF file, 0.3 MB [file spectrum.01003-22-s0001.pdf]

PCR program	Amplified region	Expected sizes	Step 1	40 times			Step 5
				Step 2	Step 3	Step 4	
(1)	*$bla_{OXA\text{-}15/464\text{-}like}$ and gyrA*	501 and 165 bp	94°C for 5mins	94°C for 30s	55°C for 30s	72°C for 40s	72°C for 5mins
				**25 times**			
(2)	(F1) gyrA	-	96°C for 1min	96°C for 10s	50°C for 5s	60°C for 4mins	4°C

Supplementary Table S1. PCR programs used

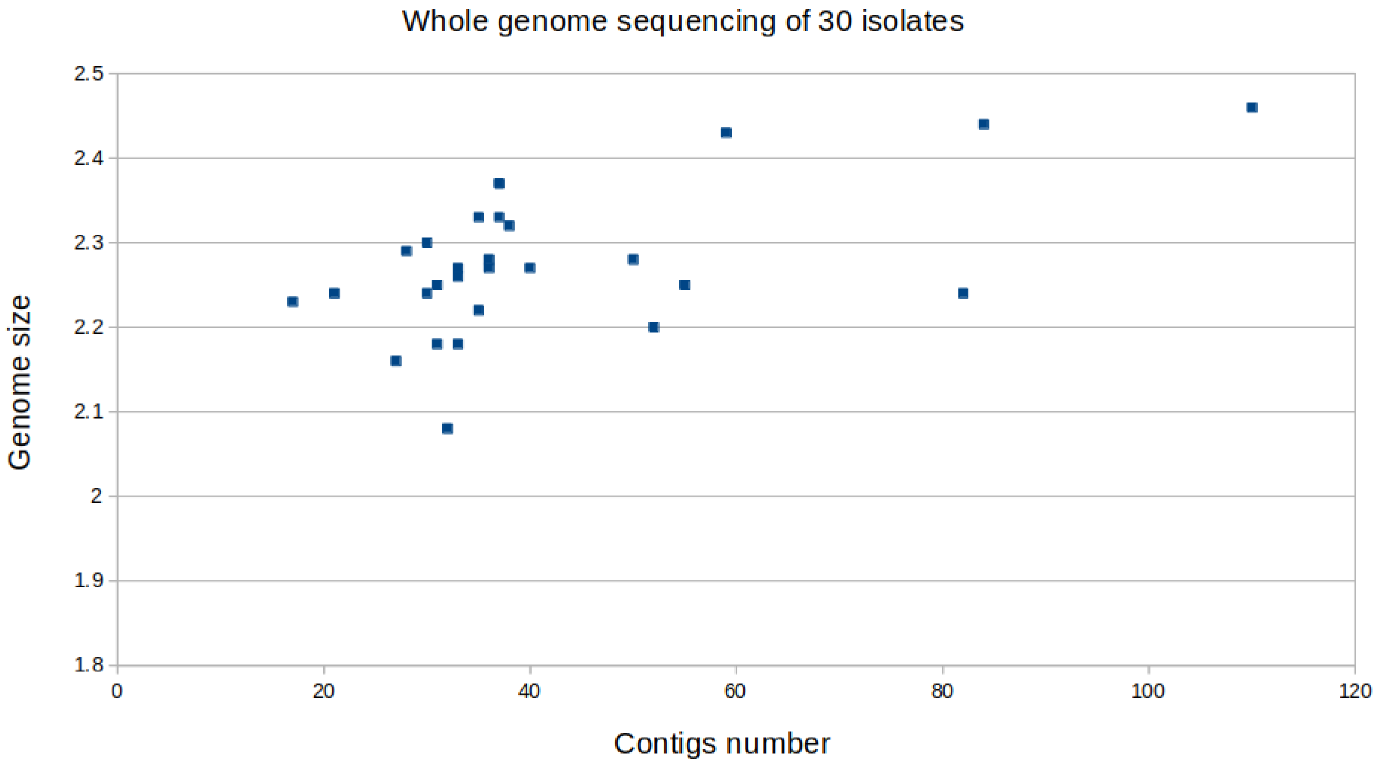

Supplementary Figure S1. Genome size (mpb) in association with contig numbers from *A. butzleri* NGS. Plot of every *A. butzleri* genome (n=30) analyzed in this study, using total size in nucleotides (y-axis) and number of unique sequences or contigs (x-axis). Reads were generated using an Illumina HiSeq 4000 sequencer from extracted DNA, and genomes were assembled using SPAdes v3.10.1. The genome sizes were in accordance with the *A. butzleri* reference genomes found in previous studies [35].

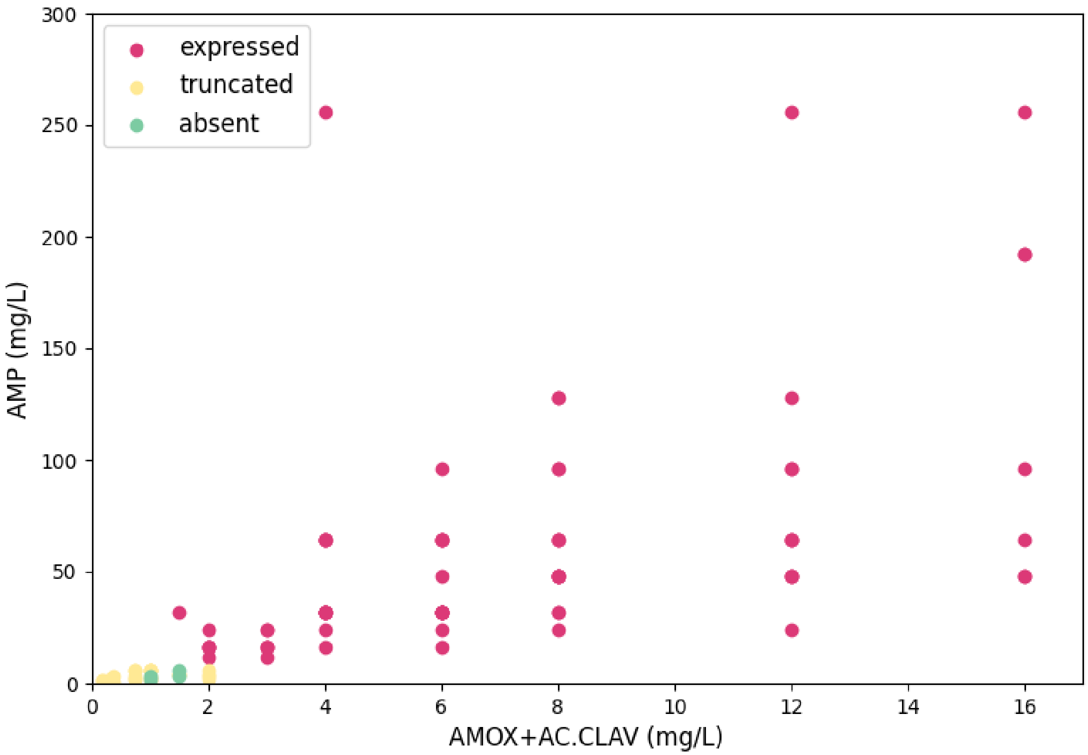

Supplementary Figure S2. MIC levels for amoxicillin and ampicillin in the presence of clavulanic acid in association with the presence of the *bla*$_{\text{OXA-15/464-like}}$. Plot graph of every *A. butzleri* isolate analyzed in this study (n=101) according to MIC levels for amoxicillin (AMOX+AC. CLAV, x-axis) and ampicillin (AMP, y-axis) and depending on *bla*$_{\textit{OXA-15/464-like}}$ sequence status: expressed (full length β-lactamase), shortened (half-size β-lactamase) or absent (no β-lactamase).

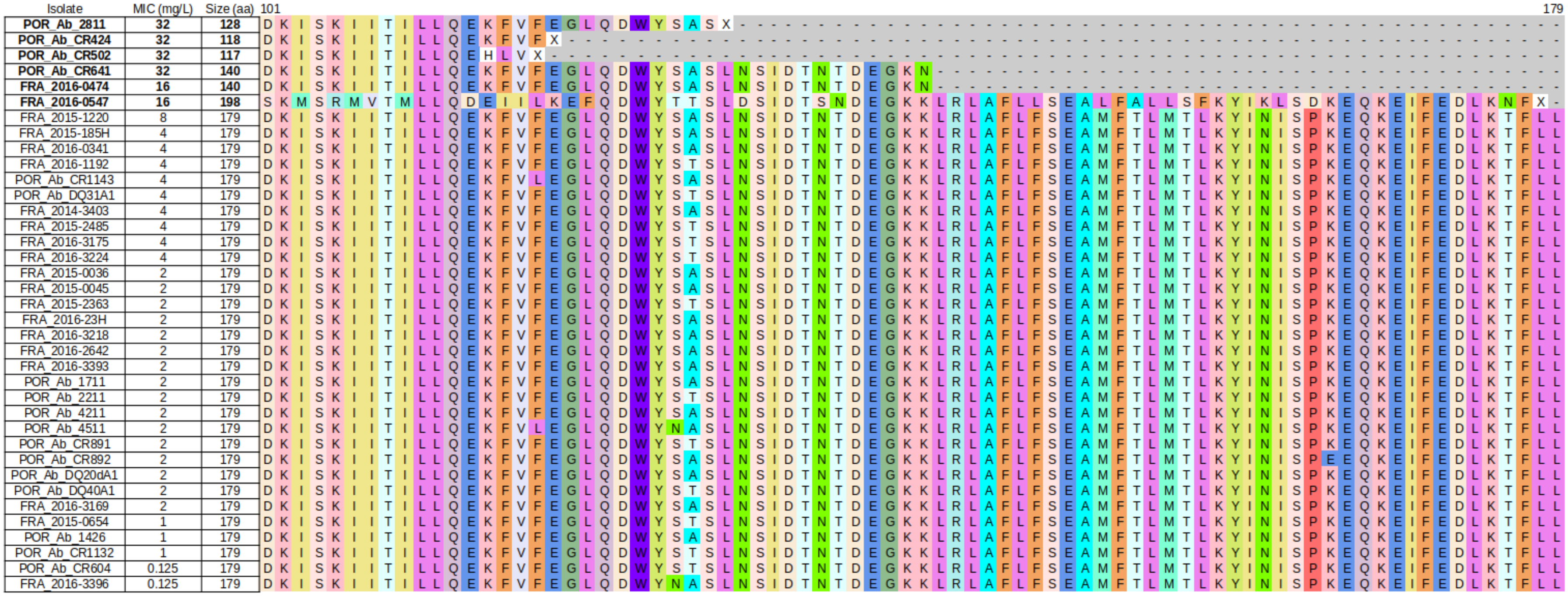

Supplementary Figure S3. TetR regulator protein sequence in association with erythromycin MIC level. TetR sequences were extracted from all *A. butzleri* isolates sequenced in this study (indexed “FRA”, n=30) and a previous study [20] from Portugal (“POR”, n=17) and are sorted by MIC values (mg/L).
